# Supplementary material for: Whole-genome view of the consequences of a population bottleneck using 2926 genome sequences from Finland and United Kingdom
Source: Eur J Hum Genet. 2017 Feb 1;25(4):477–84. doi: 10.1038/ejhg.2016.205 (PMC5346294; doi:10.1038/ejhg.2016.205)
Supplement: Supplementary Figures [file ejhg2016205x1.pdf]

Supplementary Figures

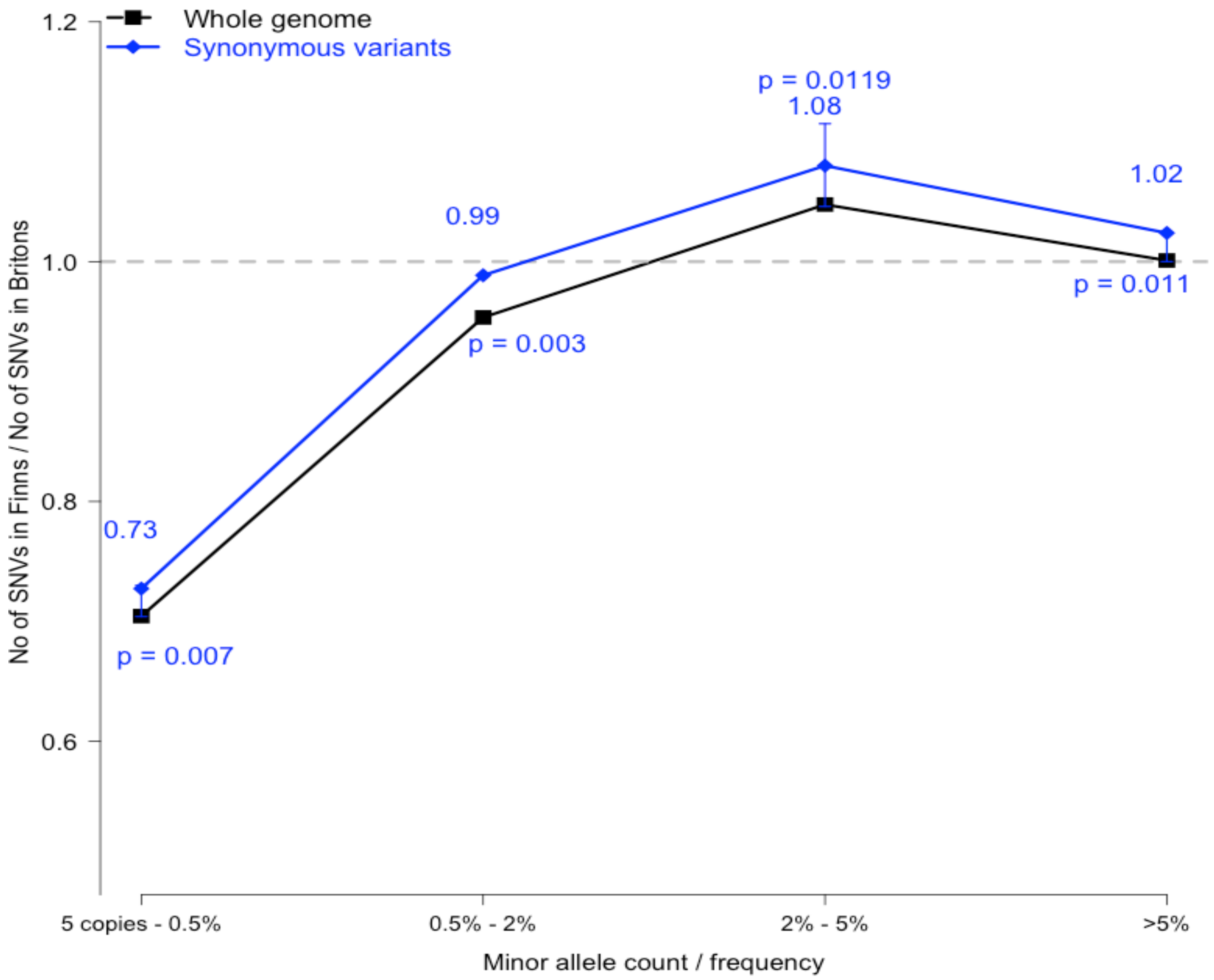

Supplementary Figure 1: Ratio of synonymous variants in Finns compared to Britons. The blue line represents the ratio of the number of synonymous SNVs in Finns compared to Britons. The black line shows the baseline enrichment observed across the whole genome.

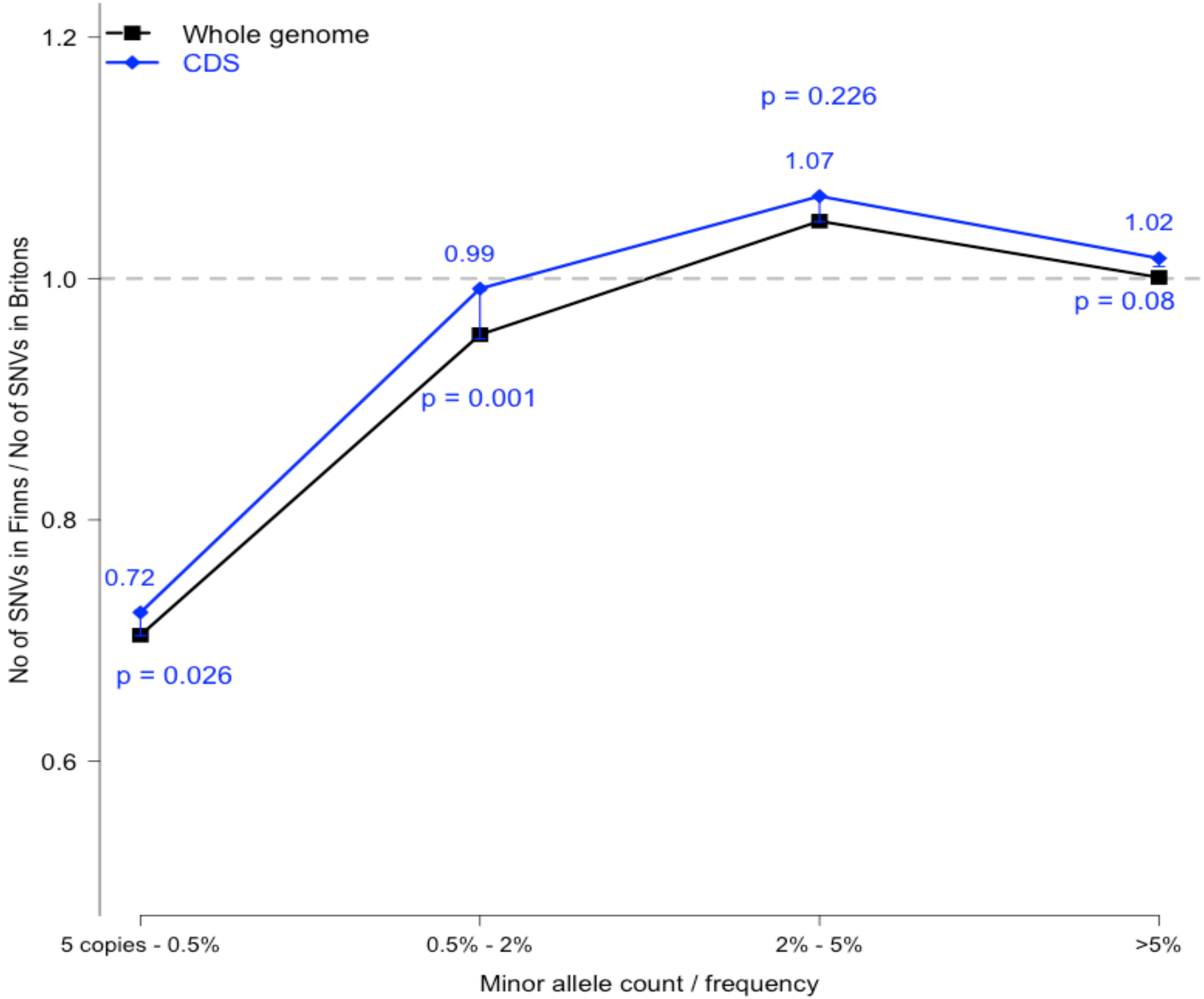

Supplementary Figure 2: Ratio of CDS variants in Finns compared to Britons. The blue line represents the ratio of the number of SNVs in the CDS regions in Finns compared to Britons. The black line shows the baseline enrichment observed across the whole genome.

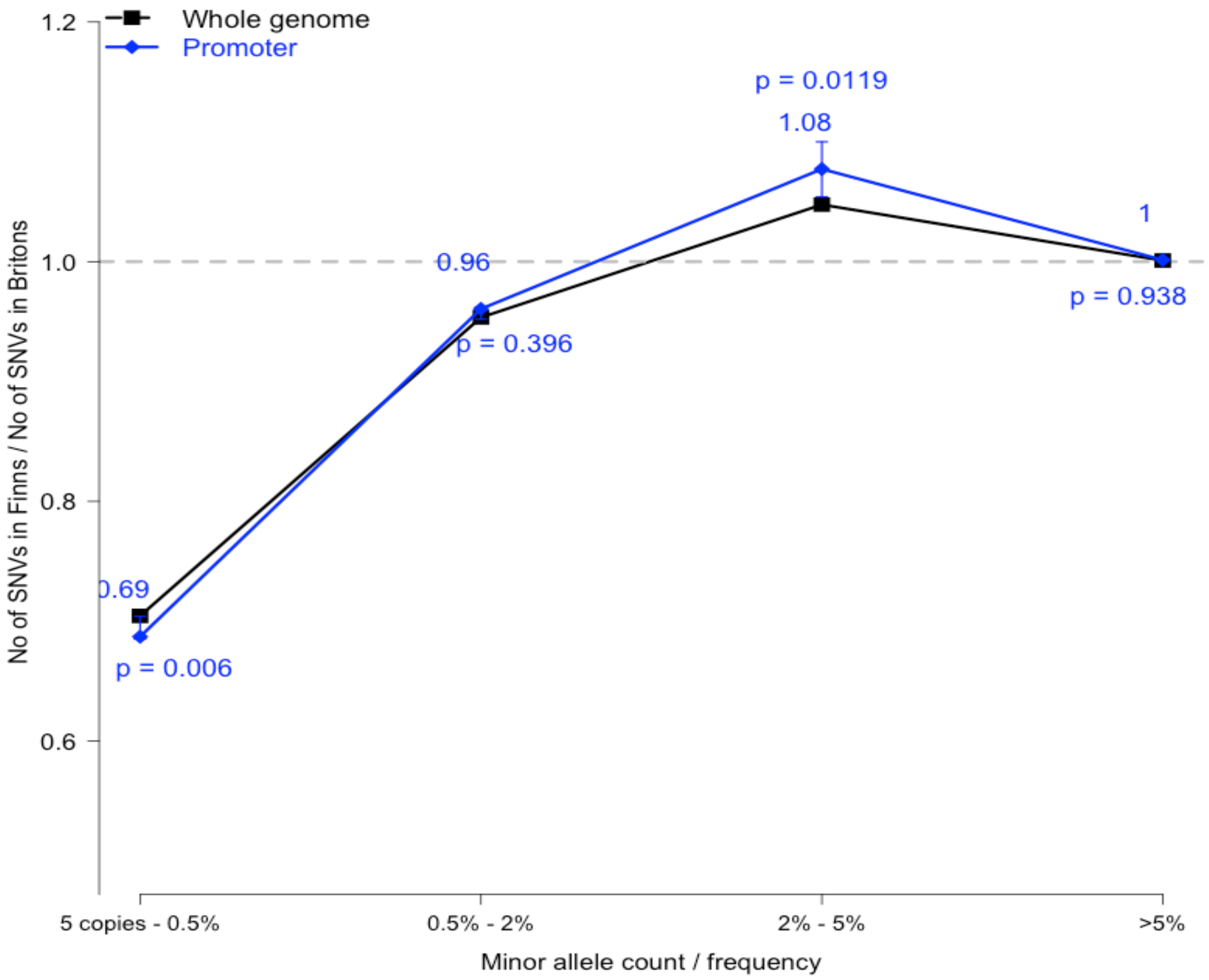

Supplementary Figure 3: Ratio of promoter variants in Finns compared to Britons. The blue line represents the ratio of the number of SNVs in the promoter regions in Finns compared to Britons. The black line shows the baseline enrichment observed across the whole genome.

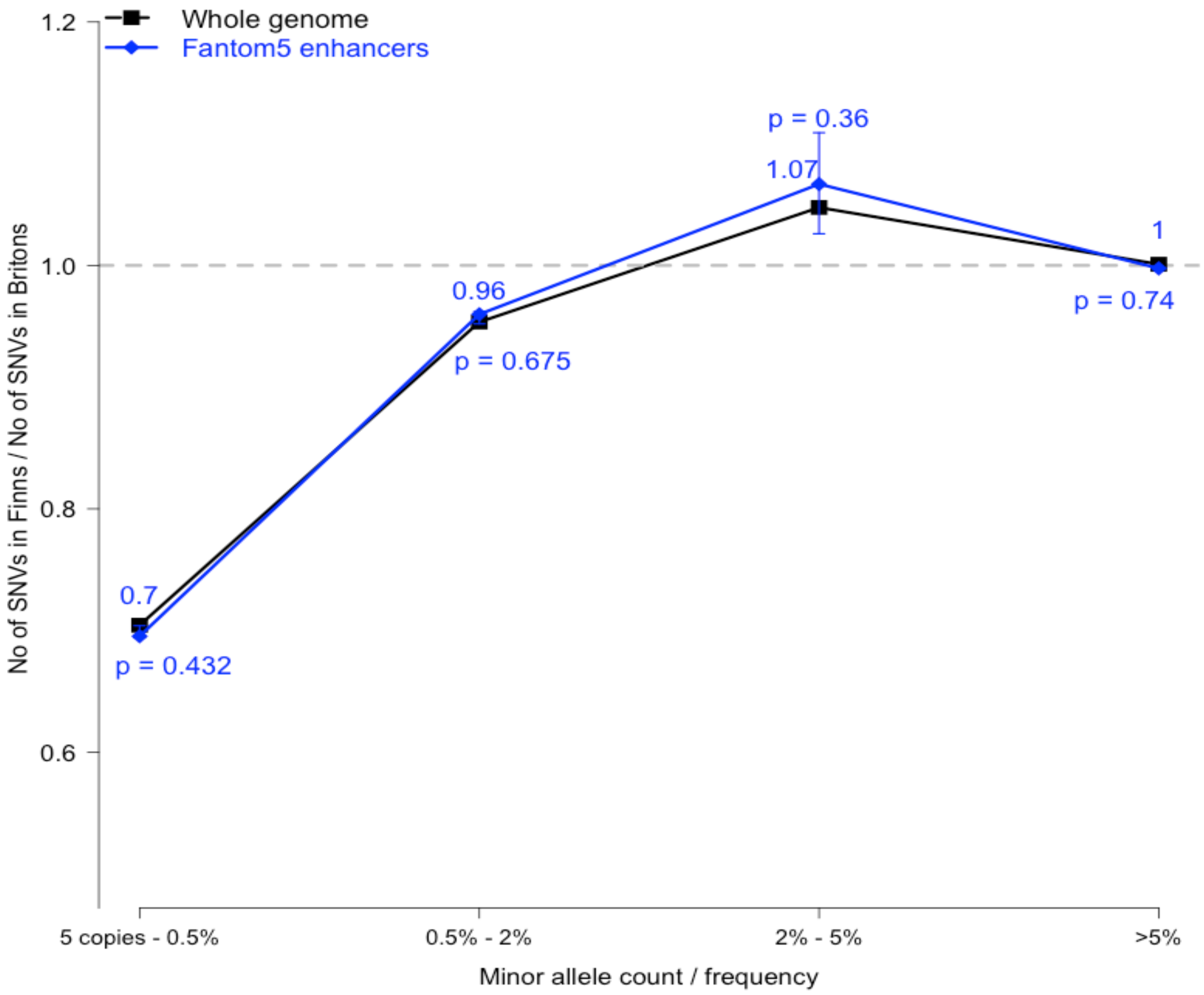

Supplementary Figure 4: Ratio of fantom5 enhancer variants in Finns compared to Britons. The blue line represents the ratio of the number of SNVs in the fantom5 enhancer regions in Finns compared to Britons. The black line shows the baseline enrichment observed across the whole genome.

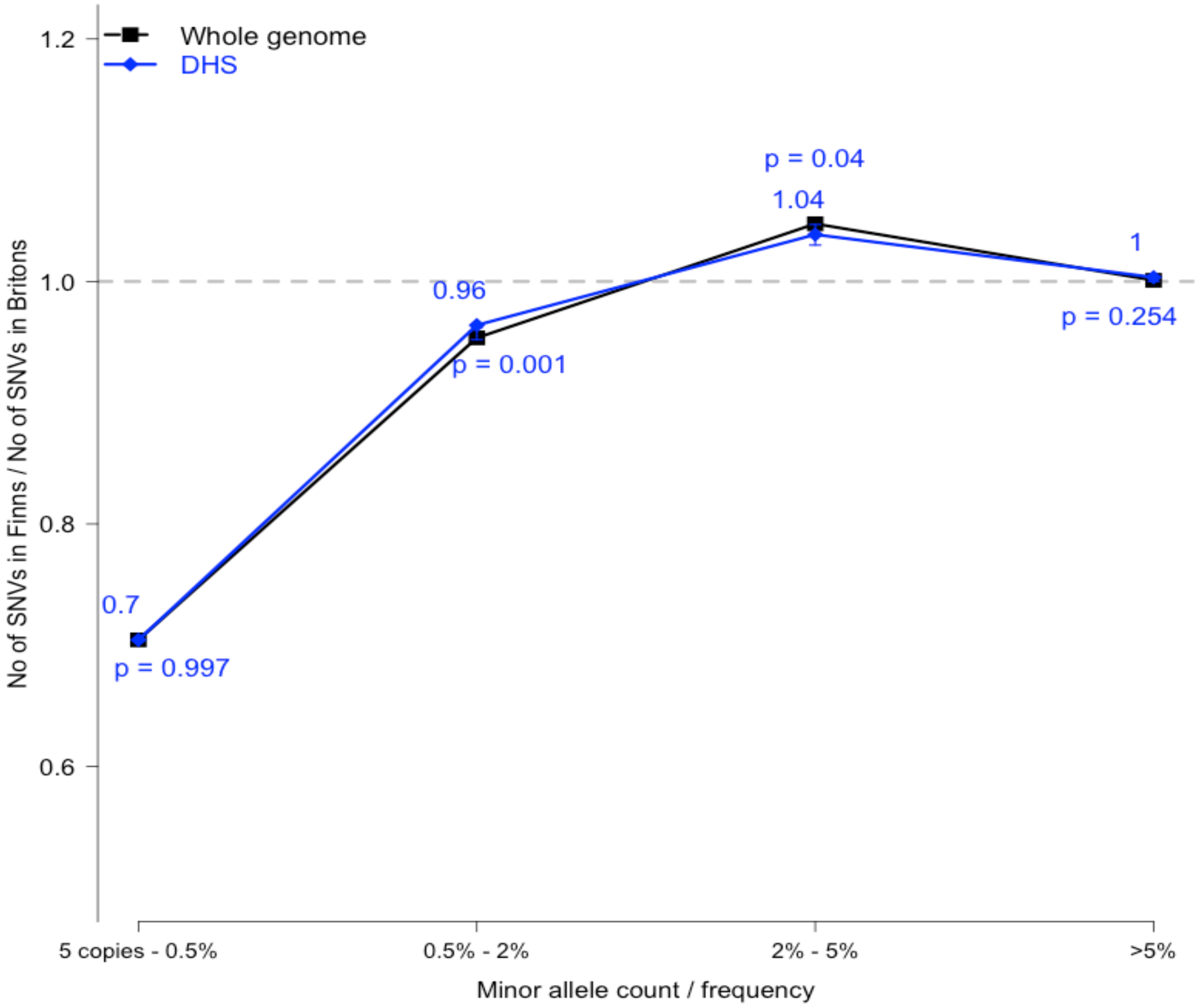

Supplementary Figure 5: Ratio of DHS variants in Finns compared to Britons. The blue line represents the ratio of the number of SNVs in the DHS regions in Finns compared to Britons. The black line shows the baseline enrichment observed across the whole genome.

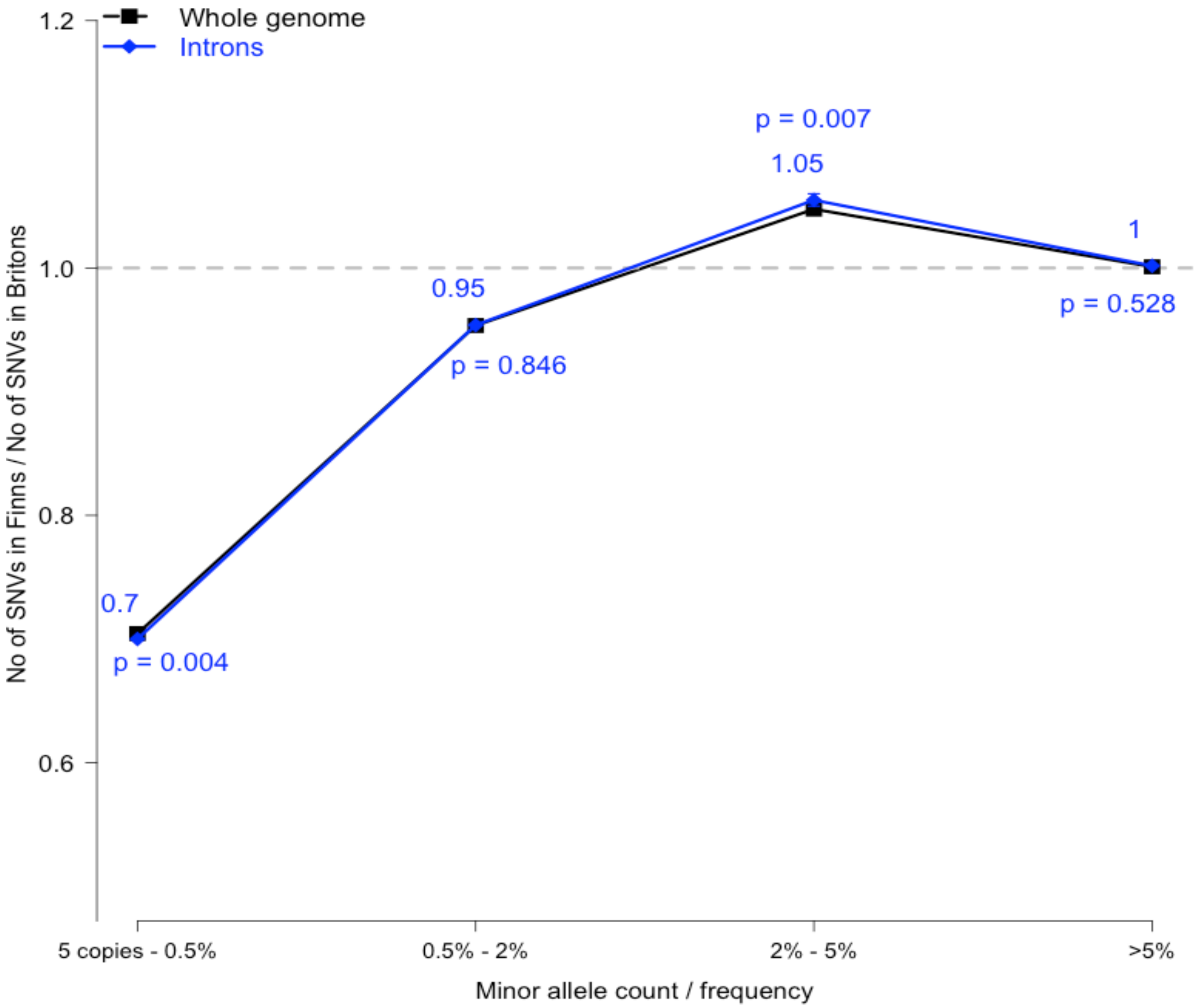

Supplementary Figure 6: Ratio of intronic variants in Finns compared to Britons. The blue line represents the ratio of the number of SNVs in the introns in Finns compared to Britons. The black line shows the baseline enrichment observed across the whole genome.

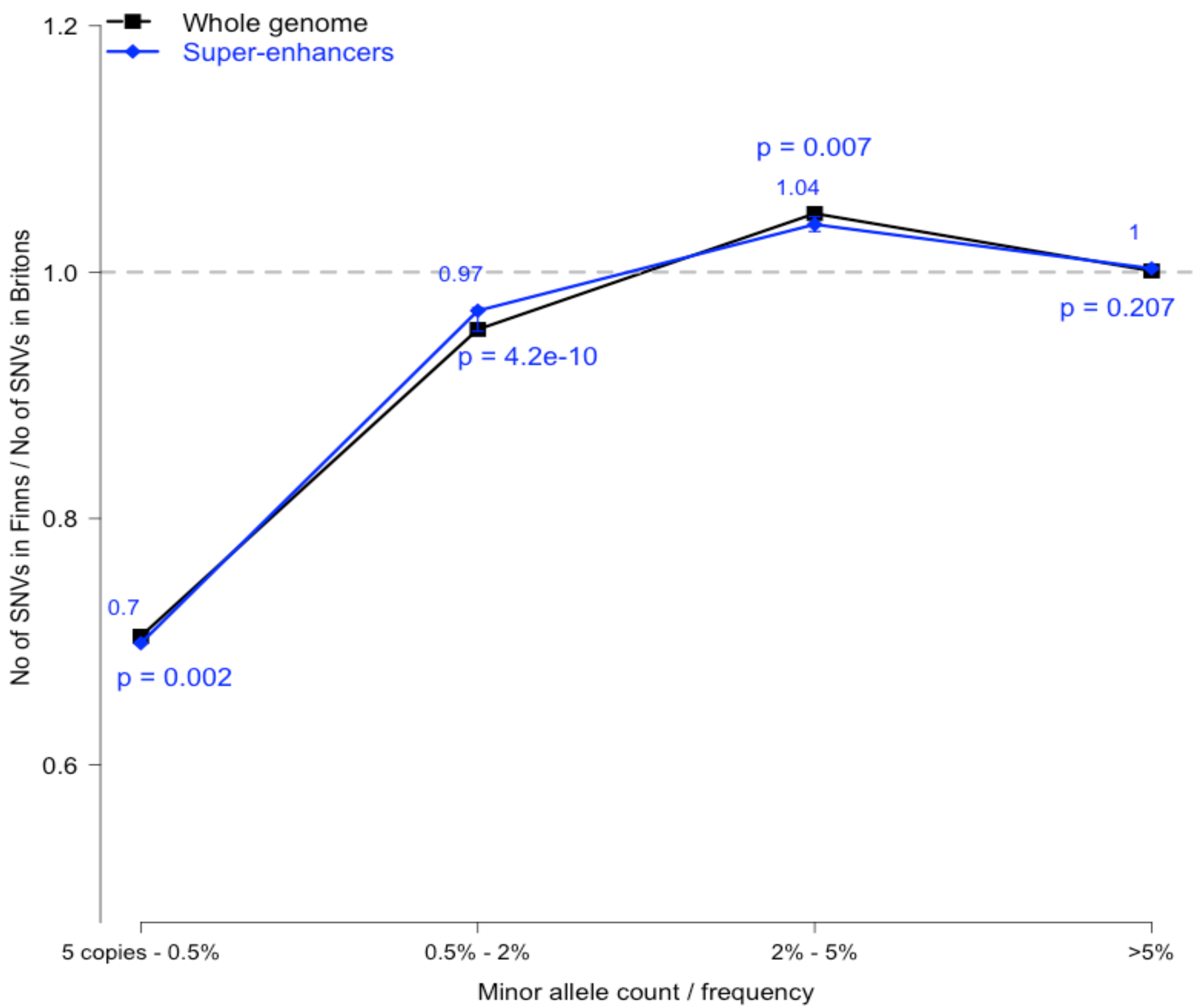

Supplementary Figure 7: Ratio of super-enhancer variants in Finns compared to Britons. The blue line represents the ratio of the number of SNVs in the super-enhancer regions in Finns compared to Britons. The black line shows the baseline enrichment observed across the whole genome.

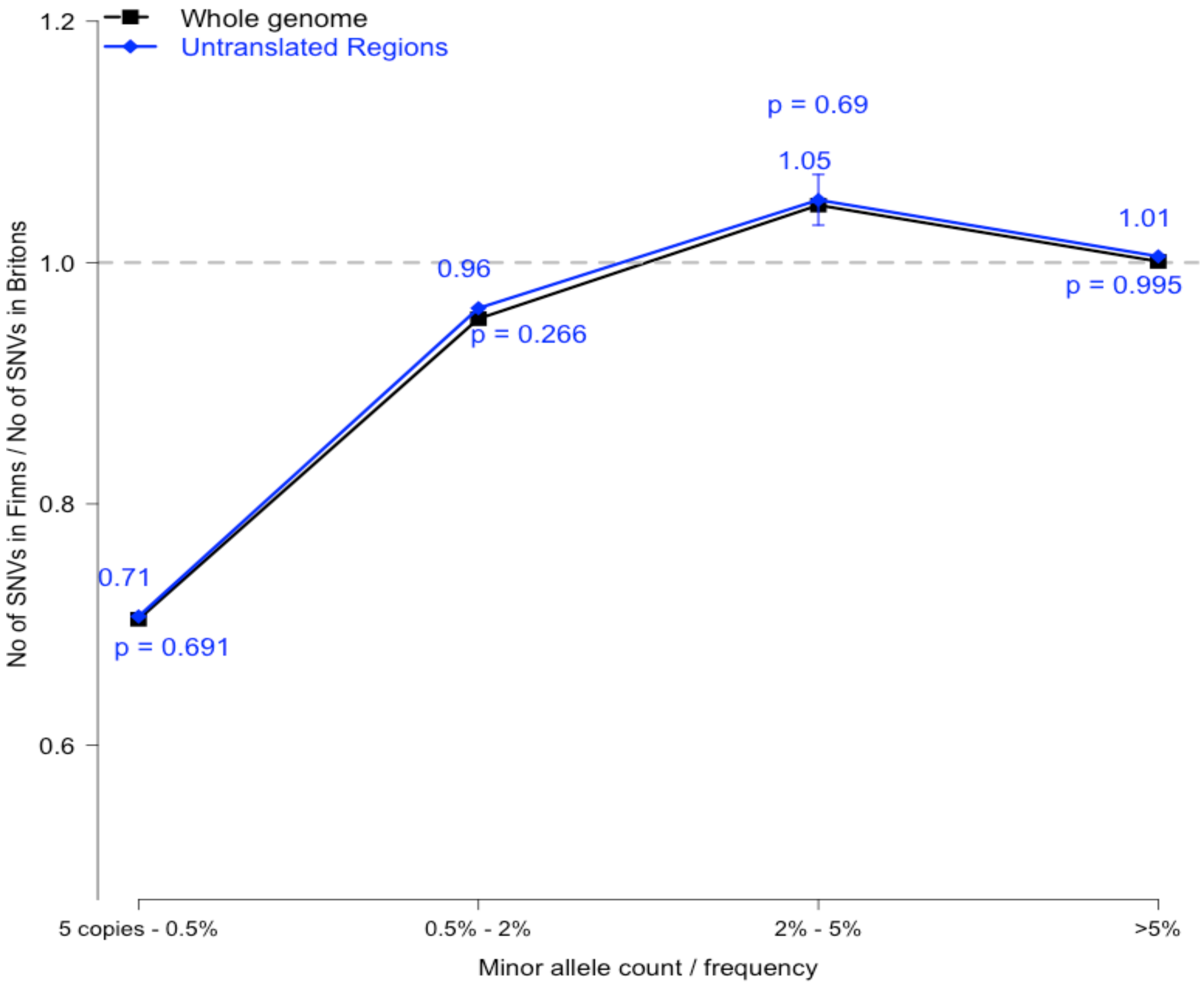

Supplementary Figure 8: Ratio of UTR variants in Finns compared to Britons. The blue line represents the ratio of the number of SNVs in the untranslated regions in Finns compared to Britons. The black line shows the baseline enrichment observed across the whole genome.

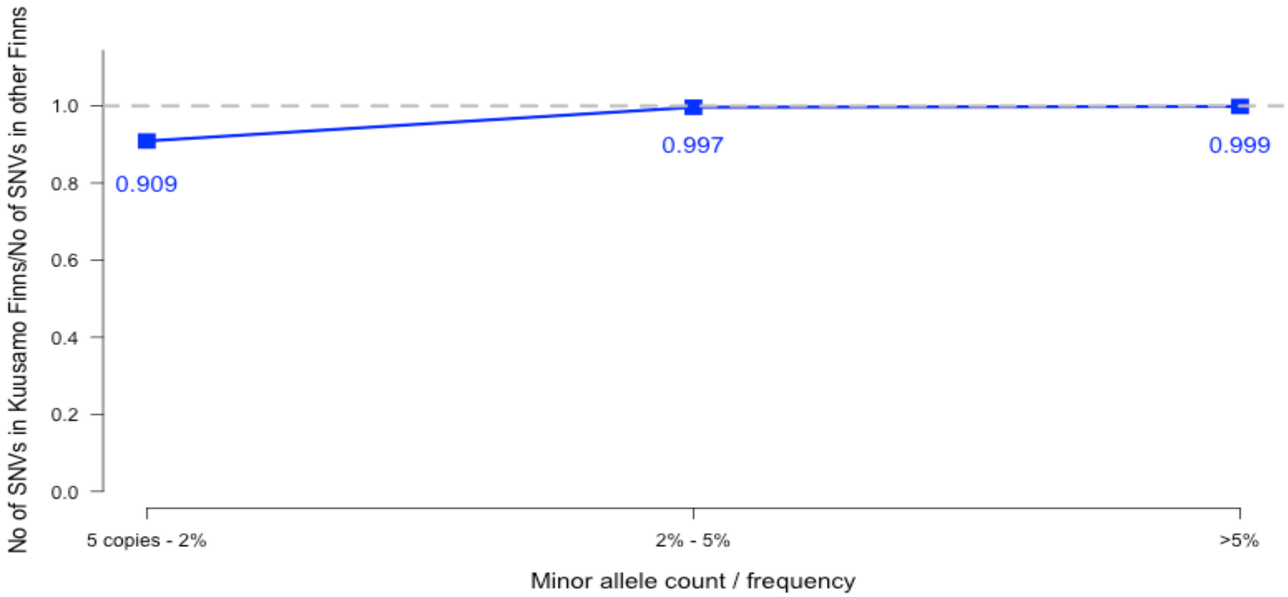

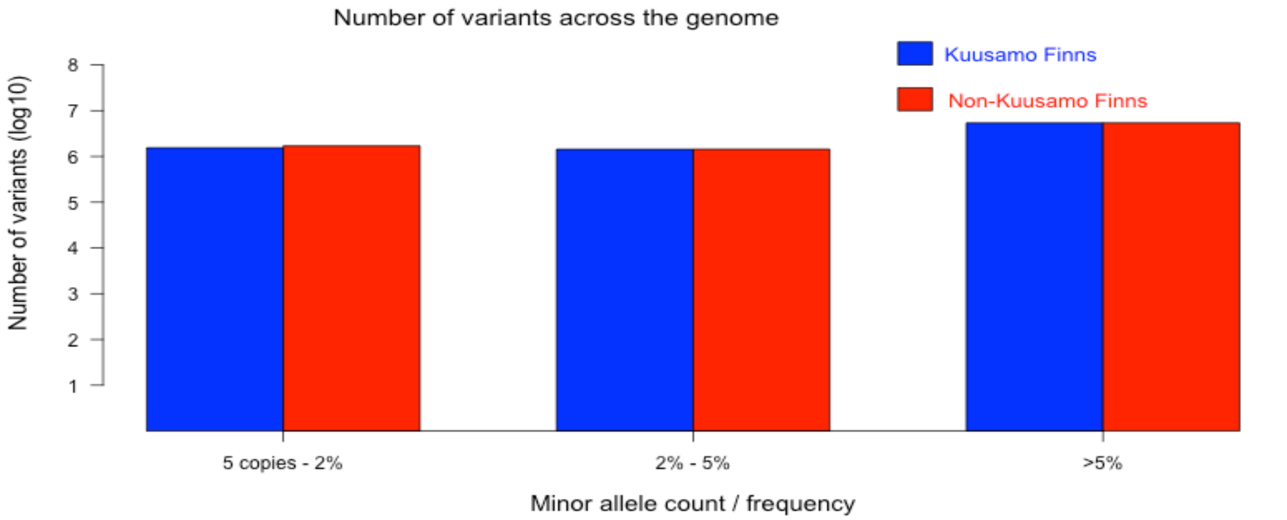

Supplementary Figure 9: (A) Ratio of number of SNVs in Kuusamo Finns compared to other Finns (non-Kuusamo). The blue line represents the ratio of the number of all SNVs across the genome. (B) The blue columns show the log10 number of variants in each minor allele frequency bin for Kuusamo Finns and the red columns show the log10 number of variants for other Finns.

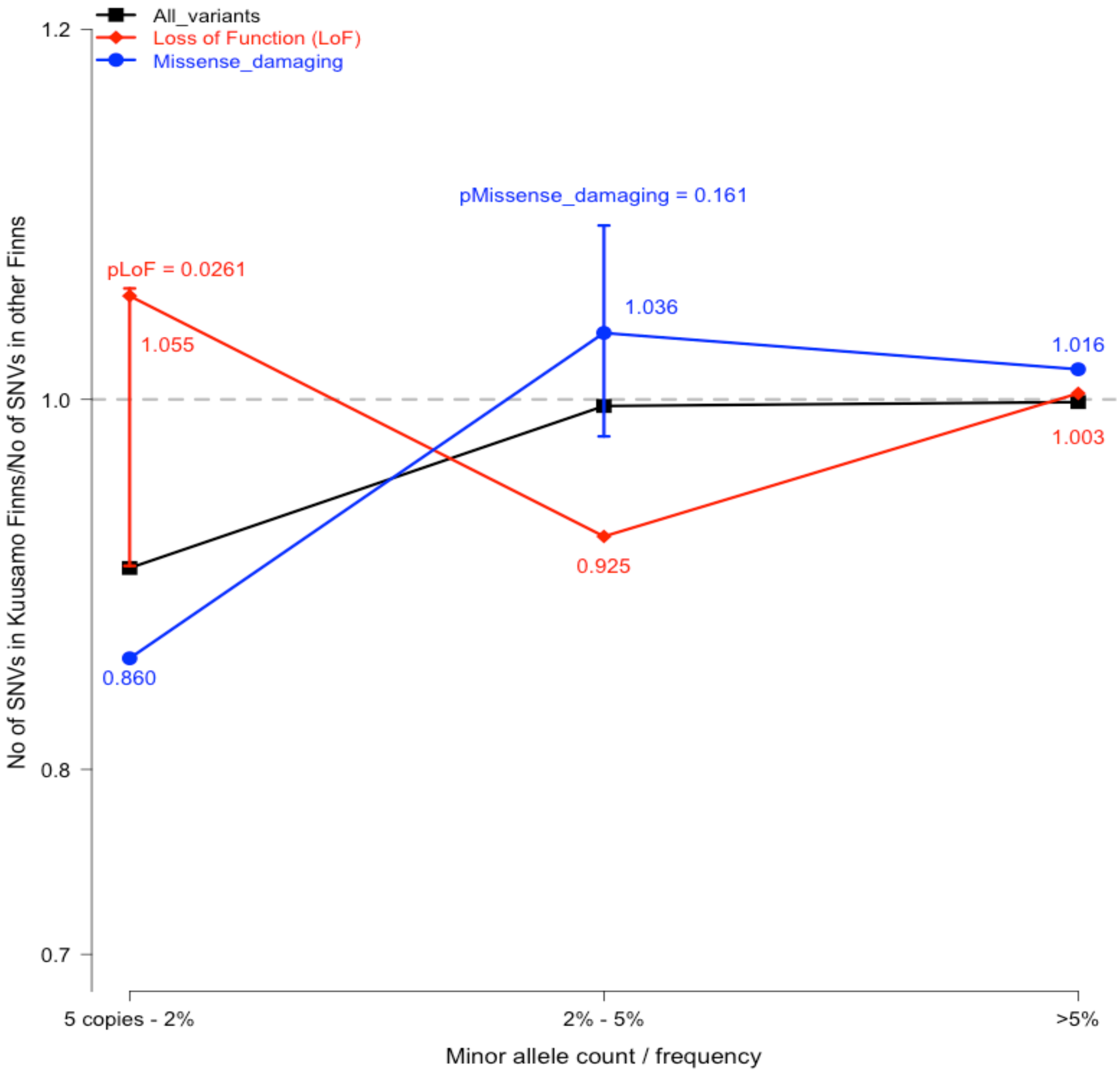

Supplementary Figure 10: Ratio of LoF and missense damaging SNVs in Kuusamo Finns compared to other Finns. The red line represents the ratio of the number of LoF variants and the blue line represents the ratio of missense damaging variants. The black line shows the baseline enrichment observed across the whole genome.

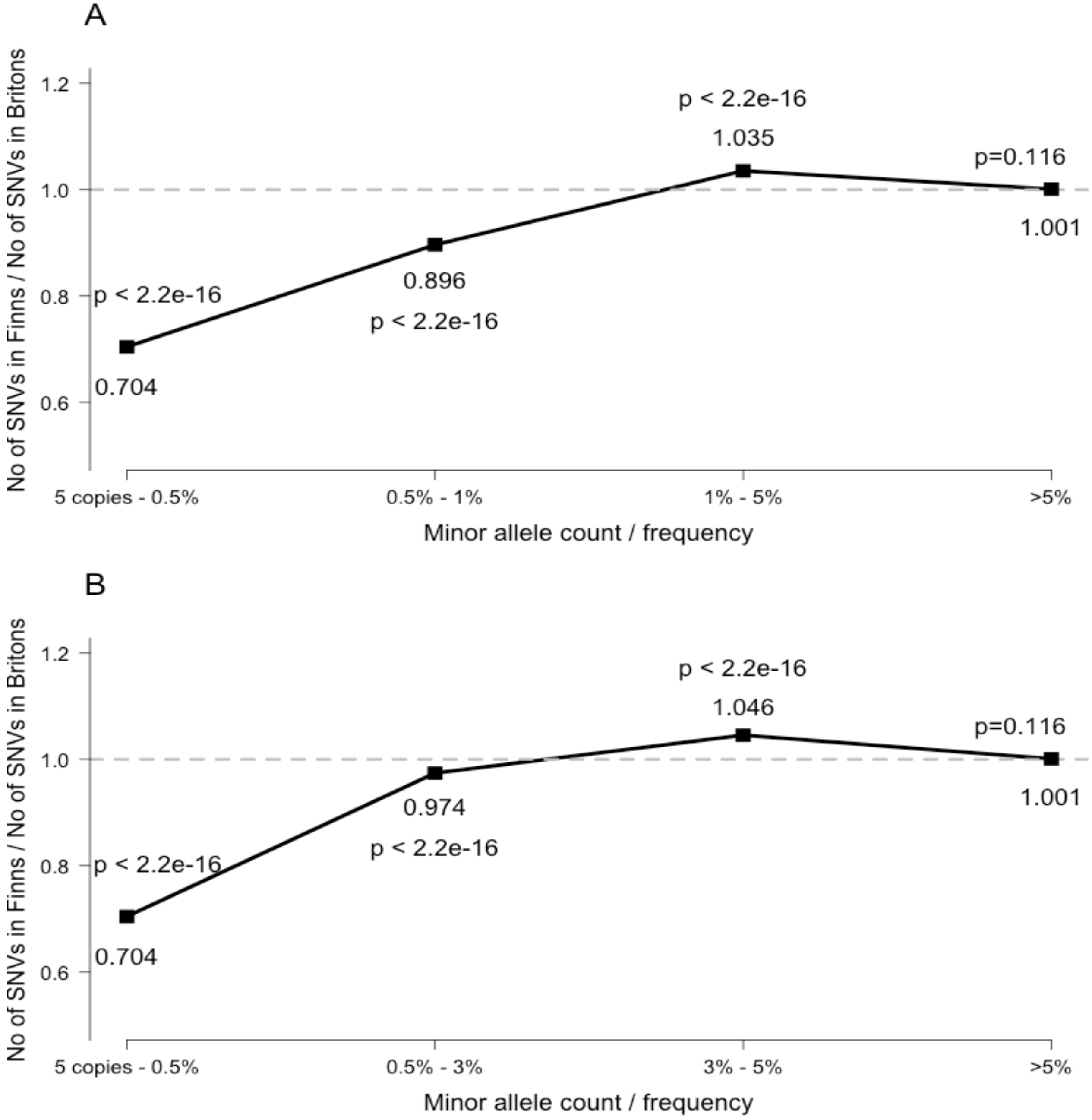

Supplementary Figure 11: (A) Allele frequency spectrum of variants across the whole genome in Finns compared to the Britons. The black line represents the ratio of the number of variants observed in Finns to those in Britons showing significant enrichment in SNVs with MAF 1 - 5%. (B) Similar to plot A but showing significant enrichment in SNVs with MAF 3 – 5%.
